# RETRACTION: Licoflavone A Suppresses Gastric Cancer Growth and Metastasis by Blocking the VEGFR-2 Signaling Pathway

**DOI:** 10.1155/jo/9803215

**Published:** 2025-09-09

**Authors:** Journal of Oncology

RETRACTION: G. Hongxia, J. Xiaojie, L. Guangxian et al., “Licoflavone A Suppresses Gastric Cancer Growth and Metastasis by Blocking the VEGFR-2 Signaling Pathway,” *Journal of Oncology* 2022 (2022): 5497991, https://doi.org/10.1155/2022/5497991.

The above article, published online on 25 April 2022 in Wiley Online Library (https://wileyonlinelibrary.com), has been retracted by John Wiley & Sons Ltd. The retraction has been agreed due to significant errors identified in Figure 5(a) where there are unexpectedly repeated elements between the VEGF 0 ng/mL + LA0µM panel and the VEGF 20 ng/mL + LA0µM panel.

This is in addition to concerns initially identified by the authors as follows:

• In Figure 2(g), the binding affinity value for LA and VEGFR-2 is incorrect.
• In Figures 7(b) and 7(c), the *y*-axis labels are incorrect.

The authors agree to the retraction.

